# Inflammation in the Female Pelvis Due to Infection from Without*Read at Gross Medical Club, Buffalo, N. Y.

**Published:** 1918-04

**Authors:** J. Henry Dowd

**Affiliations:** Buffalo, N. Y.


### Inflammation in the Female Pelvis Due to Infection From Without.*

By J. HENRY DOWD, M. D., Buffalo, N. Y.

Statistics state that 85 per cent. of all diseases of the female generative organs, that 95 per cent. of all the ovaries removed and that 45 per cent. of all operations on the pelvic organs are due to gonorrhoeal infection. With the above percentages the writer would agree, but takes exception to the infectious bacteria.

Of one thing we are quite sure, it has been established that the infection follows sexual intercourse. But, as to the gonococci being the sole and only cause, such is only a fact in a small proportion of cases.

It must be granted, because it has been proven that in every case of gonorrhoeal infection in the male two varieties of bacteria are found—the gonococci, and the strepto or straphylococci; in other words, gonorrhoea is a mixed infection.

One other fact has been proven,—gonococci are bacteria that have a specific life history on the same soil. More explicitly speaking,—gonococci born on a given soil, for a time procreate and thrive; but the day soon arrives, when, if they are not transplanted to a new medium, they lose vigor, become weaklings and die; the race in the infected individual becomes extinct. No specific date can be given as to when this change from occuous to inoccuous takes place, but it is safe to say that if a patient has been rationally treated and the soil restored to as near a normal condition as possible, no gonococci should be found after the fourth week.

In given cases no coitus has taken place for eight, ten, yes, even twelve weeks after infection; there was no discharge from the male, even the urine was clear, yet after intercourse infection in the female takes place. If not due to gonococci, then to what?

In fully 100 per cent. of cases of gonorrhoeal infection in the male the posterior urethra is involved and local foci of infection established. From this region 25 to 30 small ducts lead to the glandular substance of the prostate (to the acinous glands); according to recognized authorities not one of these ducts have sphincter muscles. In practically every case no barrier being thus present, it is with ease that infectious bacteria enter these ducts and are carried into the acinous glands. That gonococci are among the original invaders is not to be denied, but that they take along their

*Read at Gross Medical Club, Buffalo, N. Y.

companions, the strepto and the staphylococci is also an undeniable fact.

Another fact about this appalling condition as it occurs in women calls for explanation, that is that infection due to sexual contact is rarely seen at the vulva as in fully 95 per cent. of cases the seat of infection is at the cervix. On account of the pain (chordee with erections) or from moral impulses, the male suffering from gonorrhoea, either acute or chronic, foregoes sexual intercourse until inflammatory action has subsided or until on seeing no discharge at the meatus, or on finding the urine perfectly clear indicating the subsidence of urethral inflammation, he considers himself well. In fact, there may be no discharge and the urine may be macroscopically free of pus, yet the acinous glands may be involved to an extent that makes the possessor as infectious as though in the acute stage. Pus in the prostatic sinuses or in the acinous glands of the prostate has but two ways of escape,—either by ejaculation or by being brought forward by aid of the finger in the rectum (massage). Admitting an involvement of the glands, that the patient does not know is there, if coitus takes place he deposits the pus from them at just one place—the cervix.

We know that staphylo and streptococci are capable of producing inflammation, especially so on virgin soil and that is the soil found in the great majority of cases following marriage.

Three factors are necessary for the production of inflammation; a weakened or damaged condition of the tissues causing a lessening of resisting power, a medium on which bacteria can thrive and multiply, and, bacteria.

No one will deny that a damaged condition is produced in the surface tissues of genital organs of the female following marital relations, especially in the early days of marriage. It may go no further than a congestion, but this alone weakens the tissues and produces a medium upon which bacteria can grow. If the resisting power of the female genital tissues are well up to normal, congestion quickly rights itself. Even though pyogenic bacteria be deposited at the cervix, by aid of the Doederlein bacilli, the resisting power may prevent inflammatory infection.

Owing to the anatomical differences between the generative organs of the male and female, infection of those of the female by even acute gonorrhoea, symptoms similar to those in the male are never present. Except in a very limited number of cases ardorurinae is absent; chordee is never present; and, as to discharge, all females have more or less leucorrhoea. (As to discharge, the writer can report many cases of leucorrhoeal discharge so profuse that it was necessary for the patient to wear a napkin all the time; these females were not married, had never had intercourse, in fact were only young girls bewteen the ages of fifteen and seventeen; they were quite readily relieved by tonics, no local treatment was given, not even an examination was made.)

Broadly, but briefly, if constant douchings, abortions, miscarriages, and of course, new growths can be eliminated, inflammatory conditions in the female pelvis may be looked upon as due to infection from sexual contact, in which gonococci may be found in but a very limited number of cases. In a communication from Dr. Howard Kelly, some time ago he reported to me that "in fifty curettements for inflammatory conditions, gonococci were found but six times." But this one fact must never be forgotten that inflammation in the female pelvis in the absence of gonococci is in every way as dangerous as if they were present.

But it is upon the prevention of disease that medical men are today centering all their energies, and the old saying still remains true that "an ounce of prevention is worth a pound of cure."

Prevention of infection due to sexual relations, to which may be traced about 80 per cent. of the cases, can be summed up in a few words. Clean up all foci of infection in the male before allowing the resumption of sexual relations or before permitting marriage. This is easily accomplished; physicians must not discharge as well patients who have had gonorrhoea, or even a non-specific urethritis, unless the acinous glands of the prostate are found free of any inflammatory activity that produces pus; for wherever pus is found infectious bacteria are sure to be present.

The order of frequency of inflammatory involvement of the tissues in the female pelvis is as follows: Cervix, endometrium, tubes, ovaries, cellular tissue, vulva, bladder. Vaginitis is quite a rare condition because prevented by the inhibiting action of Doederlein bacillus, a normal inhabitant of the vaginal tract, undoubtedly placed there by nature to prevent the upward extension of inflammation from the vulva where even in the virgin strepto, staphylococci and other pathogenic bacteria are constantly found.

In the prophylaxis in the female the Doederlein bacillus comes in for attention; there is no doubt of the function of the Doederlein bacilli, then why should this protecting agent be washed away by constant and frequent douching?

It must be admitted that in inflammations of the cervix, endometrium, tubes and ovaries, solutions applied by aid of a douche cannot reach these infected parts; it is only in the presence of cellulitis, or of metritis that douches, by causing depletion, have any value whatever. When douches are necessary, the patient should be placed on her back and large quantities of the solution used as hot as is possible.

In considering the treatment only a few important points will be mentioned. In a recent suit against the American Medical Association, in which the treatment of diseases of the female generative organs played an important part, all authorities called on both sides admitted that not over ten per cent. of the cases call for operation. If we can believe these eminent authorities, strictly medical measures both local and general, must hold a very important place in the treatment and cure of these conditions.

We hear a great deal now of tissue resistance,—a most important consideration, not only in people who are ill, but also in those supposed to be in perfect health. In the latter condition, the greater the resisting power, the less the liability to infection; while in the former, the nearer to normal the resisting power the quicker resolution takes place. Resistance is furnished the tissues—the flesh, blood and bone—through the nervous system; therefore, it must be quite clear that this system should receive prompt, if not first attention, in all conditions of an inflammatory nature; this is especially true in the female who, generally speaking, is far below par. The amount of resisting power present and being used is easily and quickly determined by taking the phosphatic index; its determination will be found of the greatest value, as a guide not only in cases to be treated locally, but in those to be operated upon.

### CONCLUSIONS.

Prevent infection in the female generative organs by rendering the male non-infectious.

Before discharging as cured a patient who has had gonorrhoea, clear away all foci of infection; the acinous glands of the prostate are a seat of trouble in at least 95 per cent. of cases.

Whether the treatment be operative or simply local, in all cases raise the resisting power of the patient to as near normal as possible.

The quickest and surest way to promote resolution, is to place the diseased tissue in a condition to inhibit rather than to favor the growth of bacteria. This is quite easily accomplished by free elimination from the bowels, by hygienic measures, by tonics when necessary to raise tissue resistance and by local measures such as ichthyol ten per cent. in glycerine on a tampon applied every six or seven days, with iodin to the cervix and upper vaginal vault in alternation.

When douches are used for depletion, as in metritis or cellulitis, the patient should be placed in the recumbent position and at least one gallon of hot saline solution used.

9 North Pearl St.

---

**Pneumonia in a German Prison Camp.** Henri Mallie, Jour. de Med. de Bordeaux, Oct. 1917, gives his personal experience as a prisoner of war. In the prison camp of Alten Grabow, Prussia, between Sept. 1914 and Aug. 1, 1915 244 frank pneumonias, of almost typic course occurred among about 12,000 French and Belgian prisoners while among an equal number of Russians, 116 cases occurred. 10 of the former and 11 of the latter died. This represents an annual morbidity from pneumonia of about 22: 1000 for the Franco-Belgian group and of about 10: 1000 for the Russian. In contrast, the morbidity from the reports in time of peace was 4.08: 1000 for the French army, 5.5 for the Austrian, 6.3 for the German and 6.4 for the Italian. In war, the rarity of pneumonia has always been remarkable even in the retreat of the French in 1812 from Russia. In the war of 1870, the Germans noted the relative excess of pneumonia among French prisoners, as compared with that of their own men. Obviously, even under ideal conditions, which do not seem to prevail in the present war, prisoners are more likely to succumb to such a disease than troops on duty. (In this connection, the enormous difference in the incidence and mortality of pneumonia in different cantonments in the U. S. is noteworthy. The weekly statistics published deserve careful consideration. Camps of approximately the same population may show more pneumonia deaths in a single week in one than occur in the whole period of training from all causes, in another. Just as one is about to announce his conversion to the old view of pneumonia as a disease due to exposure, the checking of what threatens to assume the magnitude of an epidemic by a cold snap throws him to the opposite extreme of regarding the disease as practically as well as theoretically, an infectious fever and then the institution of measures designed to prevent chilling has such a beneficent action that the pendulum of opinion swings back again. Ed.)

---

**Cholesterol.** G. Luden, Jour. of Lab. and Clin. Med., Nov. 1917, compared, in 748 blood examinations, Bloor's method of quantitation with sodium ethylate as originally practiced, and his modification dispensing with this reagent. The original method always gave lower estimates, the difference being most marked in icteric blood in cases of obstructive jaundice.

